# Correction: Fluorinated aromatic PCBTF and 6:2 diPAP in bridge and traffic paints

**DOI:** 10.1039/d4em90053g

**Published:** 2024-12-09

**Authors:** Mitchell L. Kim-Fu, Ansel R. Moll, Esteban E. Hernandez, Boris Droz, Thierry N. J. Fouquet, Jennifer Field

**Affiliations:** a Department of Chemistry, Oregon State University Corvallis OR 97331 USA; b School of Chemical, Biological, and Environmental Engineering, Oregon State University Corvallis OR 97331 USA; c Department of Biological and Ecological Engineering, Oregon State University Corvallis OR 97331 USA; d Bausch + Lomb Americas Rochester NY 14609 USA; e Department of Environmental and Molecular Toxicology, Oregon State University Corvallis OR 97331 USA jennifer.field@oregonstate.edu +1-541-737-0497 +1-541-737-2265

## Abstract

Correction for ‘Fluorinated aromatic PCBTF and 6:2 diPAP in bridge and traffic paints’ by Mitchell L. Kim-Fu *et al.*, *Environ. Sci.: Processes Impacts*, 2024, https://doi.org/10.1039/D4EM00546E.

The authors regret that there was an error in the title of the manuscript and in the ‘Chemicals and solvents’ section when referring to ‘PCBTF’.

The corrected title and ‘Chemicals and solvents’ section are shown below:

**Title**: Fluorinated aromatic PCBTF and 6:2 diPAP in bridge and traffic paints

**Chemicals and solvents**

Solvents, PFAS standards of 11 target volatile PFAS [FTOHs, secondary FTOHs, perfluoroalkane sulfonamides (FASAs), and perfluoroalkane sulfonamido ethanols (FOSEs)], 55 target ionic PFAS [PFCAs, PFSAs, FASAs, perfluoroalkane sulfonamido acetic acids (FASAAs), substituted FASAs and FASAAs, fluorotelomer sulfonates, fluorotelomer carboxylic acids, diPAPs, chlorinated perfluoroalkyl ether sulfonates, perfluoroalkyl ether carboxylic acids, and cationic/zwitterionic PFAS], PCBTF (ThermoScientific, Waltham, MA), isotopically labeled extracted (surrogate) internal standards (Wellington, Guelph, ON), nonextracted internal standards, and reference fluoropolymers [PTFE, PCTFE, and Viton A] are listed in the ESI.†

The Royal Society of Chemistry apologises for these errors and any consequent inconvenience to authors and readers.

